# Early Left Ventricular Systolic and Diastolic Dysfunction in Patients with Newly Diagnosed Obstructive Sleep Apnoea and Normal Left Ventricular Ejection Fraction

**DOI:** 10.1155/2014/898746

**Published:** 2014-02-27

**Authors:** Lisulov Popovic Danica, Mirjana Krotin, Marija Zdravkovic, Ivan Soldatovic, Darko Zdravkovic, Milica Brajkovic, Vera Gardijan, Jelena Saric, Ruzica Pokrajac, Dragan Lovic, Predrag Stevanovic, Milina Tancic Gajic, Miodrag Vukcevic

**Affiliations:** ^1^University Clinical Hospital Center Bezanijska Kosa, Faculty of Medicine, University of Belgrade, 11000 Belgrade, Serbia; ^2^Institute for Biomedical Statistics, Faculty of Medicine, University of Belgrade, 11000 Belgrade, Serbia; ^3^Clinic for Internal Disease Intermedica, 18000 Nis, Serbia; ^4^Institute for Endocrinology, Metabolic Disease and Obesity, Faculty of Medicine, University of Belgrade, 11000 Belgrade, Serbia

## Abstract

The aim of the study was to evaluate whether obstructive sleep apnea (OSA) contributes directly to left ventricular (LV) diastolic and regional systolic dysfunction in newly diagnosed OSA with normal left ventricle ejection fraction. *Methods*. 125 consecutive patients were prospectively enrolled in the study. Control group consisted of 78 asymptomatic age-matched healthy subjects who did not have any cardiovascular and respiratory diseases. All patients had undergone overnight polysomnography and standard transthoracic and tissue Doppler imaging echocardiogram. *Results*. The *E/A* ratio and the peak *E* wave at mitral flow were significantly lower and the peak *A* wave at mitral flow was significantly higher in OSA patients compared with control subjects. Left ventricle isovolumetric relaxation time (IVRT) and mitral valve flow propagation (MVFP) were significantly longer in OSA patients than in controls. Tissue Doppler derived *S*′ amplitude of lateral part at mitral valve (*S*′Lm) and *E*′ wave amplitudes both at the lateral (*E*′Lm) and septal parts of the mitral valve (*E*′Sm) were significantly lower in OSA patients compared to controls. *Conclusion*. Newly diagnosed OSA patients with normal global LV function have significantly impaired diastolic function and regional longitudinal systolic function. OSA is independently associated with these changes in LV function.

## 1. Introduction

Obstructive sleep apnoea (OSA) is a common condition affecting about 5–15% of adult population in developed countries. Prevalence is increasing with age, obesity, and chronic diseases [1, 2]. The main characteristics are repetitive apnoea or hypopnoea induced by narrowing of the upper airways during sleep. Hypoxia and hypercapnia due to repetitive obstructive apnoea consequently affect the balance in myocardial oxygen demand and supply. The result is consequent development of myocardial ischaemia and compensatory activation of sympathetic nervous system. The cardiovascular involvement is an increase in left ventricular (LV) afterload and a decrease in LV preload [2, 3]. There are lots of studies demonstrating correlation of OSA and increased cardiovascular morbidity and mortality; furthermore, OSA is regarded as an independent risk for overall and cardiovascular morbidity and mortality [4–6]. On the other hand, patients with OSA frequently have a great number of standard well-known cardiovascular risk factors.

Manifested heart failure is the worst consequence of the left ventricular dysfunction caused by OSA. However, most of the patients with OSA without other cardiovascular disease usually exhibit normal LV ejection fraction (EF); nevertheless, they still may have clinical signs of LV systolic dysfunction. This is due to the fact that normal value of LVEF does not always mean normal LV systolic function. On the other hand, diastolic function is often impaired in OSA [7–9]. Myocardial ischemia and oxidative stress are the pathophysiological explanations of these disturbances [10, 11]. Left ventricular systolic function is generated by radial and longitudinal shortening and both processes are very important. Radial shortening is predominantly dependent on the contraction of circumferential myocardial fibres in the midwall, more resistant to ischemia. However, longitudinal shortening is generated by both longitudinal subendocardial and subepicardial fibres, where subendocardium is more vulnerable to myocardial ischaemia. Hence, assessment of LV longitudinal function might be a sensitive marker for detecting subclinical alterations in LV systolic performance and the early detection of the left ventricular systolic dysfunction [7, 12].

Mitral annular velocity evaluated by tissue Doppler imaging (TDI) is a sensitive marker of the LV longitudinal dysfunction. We assumed that early left ventricular systolic and diastolic dysfunctions are present in patients with newly diagnosed OSA, even when left ventricle ejection fraction is normal. Thus, the aim of the study was to evaluate whether obstructive sleep apnea (OSA) contributes directly to left ventricular (LV) diastolic and regional systolic dysfunction in newly diagnosed OSA with normal left ventricle ejection fraction.

## 2. Materials and Methods

### 2.1. Design and Settings

A total of 792 consecutive patients who had undergone overnight polysomnography (PSG), with newly diagnosed OSA at the Center for Sleep Medicine, Bezanijska kosa, from January 2011 to January 2013, were enrolled in the study. Among them, the patients who met the following conditions that might have had an influence on the results of echocardiographic parameters were excluded: (1) central sleep apnoea, (2) history of coronary artery disease or electrocardiographic changes suggestive of myocardial infarction, (3) proved myocardial ischaemia at the ergometry testing, (4) global LV systolic dysfunction (LVEF < 50%) or a history of congestive heart failure, (5) diabetes mellitus, (6) moderate to severe valvular heart diseases, (7) hypertrophic cardiomyopathy, (8) history of chronic obstructive pulmonary disease or asthma, (9) atrial fibrillation, (10) previous diagnosis of OSA and/or the previous use of continuous positive airway pressure therapy, and (11) chronic renal impairment (serum creatinine more than 112 *μ*mol/L), including chronic hemodialysispatients who did not have OSA (i.e., <5 events/h of apnea hypopnea index (AHI)). All patients had complained of daytime sleepiness and/or loud snoring. The investigation was done to the principles enumerated in the Helsinki Declaration of 1975 and informed consent was obtained after full explanation of the procedure. Finally, 125 patients (mean age: 58 ± 11 years, minimal 21, and maximal 75) were enrolled in the study.

In addition, a total of age matched 78 asymptomatic healthy subjects who did not have any cardiovascular and respiratory symptoms, volunteer to participate in study, were enrolled in study as a control group. All participants from the control group had neither hypertension nor coronary artery disease, and all had normal ergometry testing and normal arterial tension during three repeated measurements.

All patients had undergone overnight polysomnography to determine the presence and severity of OSA. Complete transthoracic echocardiogram had been also performed next morning. The two investigated groups were age matched, considering aging bias in left ventricle diastolic dysfunction. The study protocol was approved by the Ethics Committee of University of Belgrade and all subjects gave written informed consent for the enrollment.

### 2.2. Sleep Study

Overnight fully attended PSG monitoring was performed with the Alice 4 Sleep System (Respironics Inc., Murrysville, PA, USA) in the referral Center for Sleep Medicine using standard recording techniques and precise protocol of PSG monitoring [13]. Surface electrodes were applied to perform electroencephalogram, chin electromyogram, electrocardiogram, and electrooculography. Airflow was monitored using an air pressure sensor placed at the nose and thermistor placed at the nose and mouth, and arterial oxygen saturation (SaO_2_) was recorded continuously with a pulse oximeter. Apnea was defined as the disappearance of airflow for over 10 s; hypopnea was defined as a 50% or greater decrease in airflow lasting for more than 10 s associated with arousal or a 3% or greater decrease in arterial oxygen saturation from the baseline level. The AHI was calculated as the total number of apnea and hypopnea episodes per hour of sleep. According to the AHI, the patient was classified as having OSA when the obstructive component was over 5 events per hour. Severe OSA was classifies as AHI > 30 events/h [14].

### 2.3. Standard Echocardiography

Echocardiography was performed in the left lateral decubitus position with a commercially available ultrasound machine (Vivid VII, GE Healthcare, Milwaukee, WI, USA) and 3S-RS (3.5 MHz) probe. All images were obtained from standard parasternal and apical position using 2D, M-mode, and Doppler echocardiographic techniques. All examinations were performed by an experienced cardiologist who was blind for the results of polysomnography. The LV end-diastolic and end-systolic dimensions, as well as interventricular septum and posterior wall thicknesses, were obtained from M-mode echocardiography [15]. LVEF was measured using biplane Simpson's method according to the recommendation of European Association for Echocardiography [16]. Right ventricle dimension (RV) and right ventricular fractional area change (FAC RV) were also measured.

Pulse Doppler sample volume was placed at the mitral valve tips in the apical four-chamber view to record LV inflow velocity. From LV inflow velocity, early diastolic peak flow velocity (*E*), late diastolic peak flow velocity (*A*), *E*/*A* ratio, and deceleration time of the *E* wave velocity were measured and then calculated from three consecutive cardiac cycles [15]. Isovolumic contraction and relaxation times were also calculated from LV inflow and outflow tract velocities [16].

### 2.4. Tissue Doppler Echocardiography

Tissue Doppler echocardiography was recorded from the apical four-chamber view with the pulse-wave Doppler sample volume placed on the septal and lateral corners of the mitral annulus. Care was taken to ensure an ultrasound beam parallel to the direction of the mitral annular motion. Filters were set to exclude high-frequency signals, and gains were set to obtain clear tissue signals with minimal background noise. Peak systolic (*S*′), peak early (*E*′), and late (*A*′) diastolic mitral septum and lateral annular velocities were measured [14, 15].

### 2.5. Statistical Analysis

Data were expressed as mean values ± SD. Frequencies were expressed as percentages. Student's unpaired *t*-test was used to compare the means of the continuous variables between the group of patients with OSA and control group. Categorical variables were compared using *x* ^2^ test. Multivariate linear regression analysis was used to determine whether severe OSA was an independent parameter for early impaired LV diastolic and systolic function, after adjustment for parameters which were significantly correlated with LV tissue Doppler parameters in univariate analyses. Since hypertension and elevated BMI are intrinsically connected to OSA, avoiding OSA patients with these characteristics would not give the proper sample characteristics representing the whole OSA group, so adjustments of tissue Doppler echocardiographic parameters to BMI and hypertension have been done in order to overcome this confounding bias.

Computer calculations were performed using the SPSS computer program (Version 11.0; SPSS, Chicago, IL, USA). A *P* value of <0.05 was considered statistically significant.

## 3. Results

### 3.1. Patient Characteristics

Clinical characteristics of OSA patients and control subjects are shown in Table 1. Both groups were similar regarding to incidence of hypercholesterolemia and cholesterol level. However, OSA patients had significantly higher BMI and consequently significantly higher prevalence of hypertriglyceridemia compared with control subjects. The proportion of smokers in group of OSA was also significantly higher. Patients with OSA had significantly higher incidence of hypertension and significantly higher both systolic and diastolic arterial pressure.

The results of polysomnography are described in Table 2. According to AHI, even 81 patients (65%) had severe newly diagnosed OSA.

### 3.2. Echocardiography

Standard 2D echocardiographic measurements, left ventricular end-diastolic dimension (LVEDD), and left ventricular end-systolic dimension (LVESD) were significantly larger in patients with OSA compared to control subjects, as well as interventricular septum thickness (IVS) and posterior wall thickness (PW). There were significant differences in right ventricle dimension (RV) and right ventricular fractional area change (FAC RV). However, parameters of global left ventricular systolic function, global ejection fraction (EF), and fraction of shortening (FS) were similar in both groups (Table 3).

### 3.3. Transmitral and Tissue Doppler Imaging

The *E*/*A* ratio and the peak *E* wave at mitral flow were lower and the peak *A* wave at mitral flow was higher in OSA patients compared with control subjects. IVRT and MVFP were significantly longer in OSA patients compared to the controls. Tissue Doppler derived *S*′ amplitude of lateral part at mitral valve (*S*′Lm) and *E*′ wave amplitudes both at the lateral (*E*′Lm) and septal part of the mitral valve (*E*′Sm) were significantly lower in OSA patients compared to controls. However, although the amplitude of the *S*′ of the mitral part of the mitral valve (*S*′Sm) was also lower in OSA patients compared to controls, the difference was not significant (Table 4).

### 3.4. Multivariate Analysis for Tissue Doppler LV Parameters

Multivariate linear regression analysis revealed that presence of OSA was independently associated with the decreased values of *S*′Lm and *E*′Lm even after adjustment for hypertension and decreased values of *E*′Lm and *E*′Sm even after adjustments for hypertension and BMI, as well as after the adjustments for all possible bias (hypertension, BMI, gender, triglycerides, HDL, and LDL) (Table 5).

## 4. Discussion

The data in our cross-sectional study indicate impaired LV diastolic relaxation as well as impaired LV longitudinal systolic function in patients with newly diagnosed OSA and normal left ventricle ejection fraction. Several previous studies have shown that OSA is frequently accompanied with diastolic dysfunction [5–7]. However, patients with OSA have multiple cardiovascular risk factors and coexisting disorders that influence diastolic dysfunction such as ageing, obesity, gender, hypertension, triglycerides HDL, LDL, and diabetes. That was the reason we have done adjustments to BMI, hypertension, LDL, HDL, gender, and triglycerides in patients with OSA, trying to overcome these bias. Diabetics were not enrolled in the study. Few studies have also shown that OSA per se independently impairs LV diastolic function, regardless of obesity and hypertension [17–19].

The present study demonstrated that OSA in newly diagnosed patients independently affected both LV regional systolic and diastolic functions. Controversy exists regarding LV global and regional systolic function in OSA. According to the previous data, there is a higher proportion of heart failure in patients with OSA compared to general population. However, most of them have normal ejection fraction despite signs of the heart failure [20, 21]. Previous TDI studies have added additional data and demonstrated that OSA is accompanied by subclinical LV dysfunction in OSA patients [22, 23]. Altekin et al. [24] have also shown on a smaller group of patients with OSA, but not newly diagnosed, using speckle tracking echocardiography that, in the early stages of OSA, longitudinal systolic LV dysfunction is detected in addition to the diastolic dysfunction. In our study, newly diagnosed patients with OSA were enrolled, but since most of them had moderate and severe OSA, they certainly were not at the beginning of disease.

Impairment of the longitudinal left ventricle function despite normal LV ejection fraction in patients with newly diagnosed OSA has serious implications: longitudinal fibers, which are subendocardially located, are more susceptible to myocardial ischemia caused by the apnea-hypopnea episodes of the OSA. Normal ejection fraction is due to compensatory unchanged normal radial LV function. An increased LV wall tension and preload caused by the OSA lead to LV longitudinal systolic dysfunction and increased myocardial wall thickness is the consequence of the compensatory mechanism aiming at decreasing LV wall tension and protecting myocardial function.

As the LV hypertrophy and concentric remodeling progress, the subendocardial myocardial layer responsible for the longitudinal shortening becomes more susceptible to ischemic apoptosis and fibrous phenomena, resulting in reduced LV longitudinal shortening in the early stages of the OSA. Depressed myocardial contractile reserve in patients with obstructive sleep apnea was also proven by dobutamine stress echocardiography, supporting this pathophysiological rationale [25]. Several pathophysiological mechanism such as LV concentric hypertrophy, activated sympathetic nerve activity, and elevated central blood pressure have been proposed as the additional mechanisms of the LV diastolic dysfunction, but the present study demonstrated that newly diagnosed moderate to severe OSA impaired LV diastolic function independently of these suggested mechanisms. Furthermore, the study clearly showed that patients with newly diagnosed OSA besides impaired LV diastolic function have also impaired left ventricle longitudinal systolic function although they still have normal LV ejection fraction.

## 5. Limitations

The study has some limitations. Although patients and control subjects were age matched, significant differences of prevalence of coronary risk factors and body mass index between the two groups may have an impact on the left ventricular dysfunction. Thus, the early screening for coronary artery disease in OSA patients should be mandatory. Second, although OSA patients had severe degree of the disease, the real duration of the process could not be precisely determined.

However, the strength of the present study is twofold. First, this is the first echocardiographic study in OSA patients in Serbian population and in the same time one of the largest echocardiographic studies in OSA patients in general. Second, adjustments of echocardiographic parameters to BMI and hypertension have been successfully done.

## 6. Learning Points

- The study revealed that a greatest proportion of patients with newly diagnosed OSA has severe grade of OSA.
- In newly diagnosed patients with OSA with normal left ventricle ejection fraction, left ventricular function is severely impaired, both diastolic and systolic.
- There is a strong correlation between OSA and impaired left ventricle diastolic and regional systolic function, even after adjustments to hypertension and BMI, identifying OSA as an independent risk factor for early left ventricular diastolic and systolic dysfunction.

## Figures and Tables

**Table 1:** Clinical characteristics of OSA patients and control subjects.

	Patients (*n* = 125)	Controls (*n* = 78)	*P*
Males (*n*, %)	91 (72.8%)	36 (46.2%)	<0.001**
Age (years)	51.6 ± 10.7	48.8 ± 10.2	0.073
BMI (kg/m^2^)	31.6 ± 5.6	24.9 ± 2.8	<0.001**
BMI 30+ (*n*, %)	67 (53.6%)	5 (6.4%)	<0.001**
BSA (m^2^)	2.12 ± 0.23	1.91 ± 0.19	<0.001**
Hypertension (*n*, %)	69 (55.6%)	5 (6.4%)	<0.001**
Hypercholesterolemia (*n*, %)	94 (75.2%)	51 (65.4%)	0.132
Cholesterol (mmol/L)	6.02 ± 1.27	5.79 ± 1.13	0.187
Hypertriglyceridemia (*n*, %)	56 (44.8%)	11 (14.1%)	<0.001**
Triglycerides (mmol/L)	1.99 ± 1.20	1.28 ± 0.88	<0.001**
Low HDL (*n*, %)	35 (28.0%)	11 (14.1%)	0.021*
HDL (mmol/L)	1.25 ± 0.31	1.48 ± 0.32	<0.001**
Smoking (*n*, %)	45 (36.0%)	16 (20.5%)	0.019*

Results are presented as *n* (%) or mean ± SD.

BMI: body mass index; BSA: body surface area; HDL: high density lipoproteins.

**Means high significant, *means significant.

**Table 2:** The results of polysomnography.

Polysomnography	Mean ± SD	Median (minimum–maximum)
AHI index (*n*/h)	37.2 ± 21.7	34 (6–108.1)
Mean SaO_2_ (%)	92.1 ± 4.3	93 (75–97)
ODI index (events/h)	32.4 ± 26.5	22.7 (0.8–104.0)
The lowest SaO_2_ (%) >2 sec (%)	73.1 ± 16.1	79 (0–93)
Epworth score	12.1 ± 6.1	13 (1–26)

Results are presented as mean ± SD and median (minimum–maximum).

AHI: apnea hypopnea index; SaO_2_: oxygen saturation; ODI index: oxygen desaturation index.

**Table 3:** Echocardiographic parameters of OSA patients and control subjects.

	Patients (*n* = 125)	Controls (*n* = 78)	*P*
LVEDD (cm)	5.09 ± 0.49	4.78 ± 0.42	<0.001**
LVESD (cm)	3.21 ± 0.41	3.06 ± 0.41	<0.001**
IVSd (cm)	1.11 ± 0.14	0.97 ± 0.13	<0.001**
PWd (cm)	0.98 ± 0.13	0.85 ± 0.11	<0.001**
EF (%)	65.8 ± 6.7	65.9 ± 7.3	0.912
FS (%)	36.5 ± 5.4	36.5 ± 5.8	0.960
RV (cm)	2.37 ± 0.37	2.24 ± 0.32	0.011*
FAC RV (%)	42.03 ± 9.20	46.08 ± 11.56	0.010*

Results are presented as mean ± SD.

LVEDD: left ventricular end-diastolic diameter; LVESD: left ventricular end-systolic diameter; IVSd: interventricular thickness (diastole); PWd: left ventricular posterior wall diameter (diastole); EF: ejection fraction; FS: fraction of shortening; RV: right ventricular diameter; FAC RV: right ventricular fractional area change.

**Means high significant, *means significant.

**Table 4:** Transmitral and tissue Doppler imaging parameters of OSA patients and control subjects.

	Patients (*n* = 125)	Controls (*n* = 78)	*P*
SBP (mmHg)	131.2 ± 11.8	119.6 ± 10.7	<0.001**
DBP (mmHg)	82.1 ± 8.2	77.7 ± 6.8	<0.001**
E/A ratio	1.04 ± 0.27	1.24 ± 0.25	<0.001**
DCT (ms)	212.8 ± 59.8	198.8 ± 57.5	0.103
Peak E MV (m/s)	0.74 ± 0.15	0.79 ± 0.13	0.018*
Peak A MV (m/s)	0.76 ± 0.15	0.68 ± 0.13	<0.001**
IVRT (ms)	90.3 ± 11.8	85.9 ± 6.5	<0.001**
MVFP (cm/s)	0.043 ± 0.020	0.032 ± 0.015	<0.001**
S′Lm (cm/s)	9.30 ± 2.60	10.31 ± 2.14	0.005**
E′Lm (cm/s)	10.28 ± 3.02	13.72 ± 3.07	<0.001**
A′Lm (cm/s)	10.43 ± 2.51	9.94 ± 2.57	0.183
S′Sm (cm/s)	8.43 ± 1.75	8.81 ± 1.45	0.115
E′Sm (cm/s)	8.61 ± 2.40	10.83 ± 2.44	<0.001**
A′Sm (cm/s)	10.21 ± 2.26	10.09 ± 2.29	0.708

Results are presented as mean ± SD.

SBP: systolic blood pressure; DBP: diastolic blood pressure; DCT: deceleration time; MV: mitral valve; IVRT: isovolumic relaxation time; MVFP: mitral valve flow propagation; S′LM: tissue Doppler derived S′ amplitude of lateral part at mitral valve; E′Lm: tissue Doppler derived E′ amplitude of lateral part at mitral valve; A′Lm: tissue Doppler derived A′ amplitude of lateral part at mitral valve; S′Sm: tissue Doppler derived S′ amplitude of septal part at mitral valve; E′Sm: tissue Doppler derived E′ amplitude of septal part at mitral valve; A′Sm: tissue Doppler derived A′ amplitude of septal part at mitral valve.

**Means high significant, *means significant.

**Table 5:** Multivariate regression analysis for tissue Doppler LV parameters.

Adjustment	S′Lm	E′Lm	A′Lm	S′Sm	E′Sm	A′Sm
	Standard *β* (*P* value)					
No adjustment	−0.198 (0.005)**	−0.483 (<0.001)**	0.094 (0.183)	−0.111 (0.115)	−0.410 (<0.001)**	0.026 (0.708)
Gender	−0.260 (<0.001)**	−0.495 (<0.001)**	0.076 (0.301)	−0.162 (0.025)*	−0.398 (<0.001)**	−0.016 (0.827)
HTA	−0.223 (0.006)**	−0.405 (<0.001)**	0.074 (0.363)	−0.124 (0.129)	−0.367 (<0.001)**	0.003 (0.974)
HTA, BMI	−0.167 (0.059)	−0.390 (<0.001)**	0.093 (0.302)	−0.140 (0.120)	−0.395 (<0.001)**	0.025 (0.783)
HTA, BMI, and gender	−0.232 (0.008)**	−0.401 (<0.001)**	0.074 (0.421)	−0.186 (0.041)*	−0.381 (<0.001)**	−0.018 (0.841)
HTA,BMI, gender, and Tg	−0.225 (0.012)*	−0.402 (<0.001)**	0.051 (0.508)	−0.179 (0.053)	−0.374 (<0.001)**	−0.018 (0.845)
HTA, BMI, gender, Tg, HDL, and LDL	−0.230 (0.009)**	−0.408 (<0.001)**	0.064 (0.494)	−0.180 (0.051)	−0.375 (<0.001)**	−0.017 (0.855)

S′LM: tissue Doppler derived S′ amplitude of lateral part at mitral valve; E′Lm: tissue Doppler derived E′ amplitude of lateral part at mitral valve; A′Lm: tissue Doppler derived A′ amplitude of lateral part at mitral valve; S′Sm: tissue Doppler derived S′ amplitude of septal part at mitral valve; E′Sm: tissue Doppler derived E′ amplitude of septal part at mitral valve; A′Sm: tissue Doppler derived A′ amplitude of septal part at mitral valve; HTA: hypertension; Tg: triglycerides; HDL: high density lipoproteins; LDL: low density lipoproteins; BMI: body mass index.

**Means high significant, *means significant.

## References

[1] Fuhrman C, Fleury B, Nguyên XL, Delmas MC (2012). Symptoms of sleep apnea syndrome: high prevalence and underdiagnosis in the French population. *Sleep Medicine*.

[2] Mannarino MR, Di Filippo F, Pirro M (2012). Obstructive sleep apnea syndrome. *European Journal of Internal Medicine*.

[3] Leong WB, Arora T, Jenkinson D (2013). The prevalence and severity of obstructive sleep apnea in severe obesity: the impact of ethnicity. *Journal of Clinical Sleep Medicine*.

[4] Rich J, Raviv A, Raviv N, Brietzke SE (2012). All-cause mortality and obstructive sleep apnea severity revisited. *Otolaryngology—Head and Neck Surgery*.

[5] Zamarrón C, Valdés Cuadrado L, Alvarez-Sala R (2013). Pathophysiologic mechanisms of cardiovascular disease in obstructive sleep apnea syndrome. *Pulmonary Medicine*.

[6] Ge X, Han F, Huang Y (2013). Is obstructive sleep apnea associated with cardiovascular and all-cause mortality?. *PLoS ONE*.

[7] Butt M, Dwivedi G, Shantsila A, Khair OA, Lip GY (2012). Left ventricular systolic and diastolic function in obstructive sleep apnea: impact of continuous positive airway pressure therapy. *Circulation: Heart Failure*.

[8] Usui Y, Takata Y, Inoue Y (2013). Severe obstructive sleep apnea impairs left ventricular diastolic function in non-obese men. *Sleep Medicine*.

[9] Wachter R, Lüthje L, Klemmstein D (2013). Impact of obstructive sleep apnoea on diastolic function. *European Respiratory Journal*.

[10] Meier M, Andreas S (2012). Mechanisms of cardiovascular co-morbidity in patients with obstructive sleep apnoea syndrome. *Pneumologie*.

[11] Murri M, García-Delgado R, Alcázar-Ramírez J (2011). Effect of CPAP on oxidative stress and circulating progenitor cell levels in sleep patients with apnea-hypopnea syndrome. *Respiratory Care*.

[12] Won CH, Chun HJ, Chandra SM, Sarinas PS, Chitkara RK, Heidenreich PA (2013). Severe obstructive sleep apnea increases mortality in patients with ischemic heart disease and myocardial injury. *Sleep and Breathing*.

[13] Usui Y, Tomiyama H, Hashimoto H (2008). Plasma B-type natriuretic peptide level is associated with left ventricular hypertrophy among obstructive sleep apnoea patients. *Journal of Hypertension*.

[14] The Report of an American Academy of Sleep Medicine Task Force (1999). Sleep-related breathing disorders in adults: recommendations for syndrome definition and measurement techniques in clinical research. *Sleep*.

[15] Devereux RB (1987). Detection of left ventricular hypertrophy by M-mode echocardiography. Anatomic validation, standardization, and comparison to other methods. *Hypertension*.

[16] Galderisi M, Henein MY, Dhooge J (2011). Recommendations of the European association of echocardiography how to use echo-doppler in clinical trials: different modalities for different purposes. *European Journal of Echocardiography*.

[17] Kim SM, Cho KI, Kwon JH, Lee HG, Kim TI (2012). Impact of obstructive sleep apnea on left atrial functional and structural remodeling beyond obesity. *Journal of Cardiology*.

[18] Cho KI, Kwon JH, Kim SM, Park TJ, Lee HG, Kim TI (2012). Impact of obstructive sleep apnea on the global myocardial performance beyond obesity. *Echocardiography*.

[19] Usui Y, Takata Y, Inoue Y (2012). Coexistence of obstructive sleep apnoea and metabolic syndrome is independently associated with left ventricular hypertrophy and diastolic dysfunction. *Sleep and Breathing*.

[20] Varol E, Akcay S, Ozaydin M, Ozturk O, Cerci SS, Sahin U (2010). Influence of obstructive sleep apnea on left ventricular mass and global function: sleep apnea and myocardial performance index. *Heart and Vessels*.

[21] Arias MA, García-Río F, Alonso-Fernández A, Mediano O, Martínez I, Villamor J (2005). Obstructive sleep apnea syndrome affects left ventricular diastolic function: effects of nasal continuous positive airway pressure in men. *Circulation*.

[22] Nishikage T, Nakai H, Lang RM, Takeuchi M (2008). Subclinical left ventricular longitudinal systolic dysfunction in hypertension with no evidence of heart failure. *Circulation Journal*.

[23] Kasikcioglu HA, Karasulu L, Tartan Z, Kasikcioglu E, Cuhadaroglu C, Cam N (2007). Occult cardiac dysfunction in patients with obstructive sleep apnea syndrome revealed by tissue Doppler imaging. *International Journal of Cardiology*.

[24] Altekin RE, Yanikoglu A, Baktir AO (2012). Assessment of subclinical left ventricular dysfunction in obstructive sleep apnea patients with speckle tracking echocardiography. *The International Journal of Cardiovascular Imaging*.

[25] Okuda N, Ito T, Emura N (2007). Depressed myocardial contractile reserve in patients with obstructive sleep apnea assessed by tissue doppler imaging with dobutamine stress echocardiography. *Chest*.

